# Fractional carbon dioxide laser alone and as an assisted drug delivery for treatment of alopecia areata: a clinical, dermoscopic and immunohistochemical study

**DOI:** 10.1007/s00403-023-02565-x

**Published:** 2023-02-21

**Authors:** Azza Mahfouz Abdel Meguid, Alaa Ghazally, Asmaa M. Ahmed, Radwa M. Bakr

**Affiliations:** 1 Dermatology, Venereology and Andrology Department, Faculty of Medicine, Assiut University, Assiut, 71515 Egypt; 2 Pathology Department, Faculty of Medicine, Assiut University, Assiut, Egypt

**Keywords:** Alopecia areata, Fractional carbon dioxide laser, Triamcinolone acetonide, Platelet-rich plasma, Vitamin D3 solution, Decorin

## Abstract

Alopecia areata (AA) is a common cause of hair loss with no available universally successful treatment. Thus, new innovative treatments are urgently needed. This research aimed to evaluate the effectiveness of fractional carbon dioxide laser (FCL) alone or combined with triamcinolone acetonide (TA) solution, platelet-rich plasma (PRP), or vitamin D3 solution in treating AA. Sixty-four AA patients with 185 lesions were recruited and divided into four treatment groups. All patients received FCL either alone (group A, *n* = 19) or followed by topical TA (group B, *n* = 16) or PRP (group C, *n* = 15), or vitamin D3 solution (group D, *n* = 14). The response was assessed using Alopecia Areata Severity Index (AASI), MacDonald Hull and Norris grading, and trichoscopy. Histopathological features and immunohistochemical decorin expression were studied. All groups showed significant improvement in AASI compared to the baseline, with insignificant differences between them. Post-treatment, trichoscopic features of disease activity significantly decreased in all groups. Compared to control biopsies, both anagen follicles and decorin expression were significantly decreased in all pretreatment specimens. After treatment, all groups showed significantly increased anagen follicles and decorin expression compared to the baseline. Accordingly, FCL is an effective treatment for AA alone or combined with TA, PRP, or vitamin D3 solution. In AA, Decorin expression was downregulated, while enhanced expression following successful treatment occurred. This suggests the role of decorin in AA pathogenesis. However, further research is still recommended to clarify the exact role of decorin in AA pathogenesis and to investigate the therapeutic benefits of decorin-based therapy.

## Introduction

Alopecia areata (AA) is one of the most common causes of non-scarring alopecia affecting the scalp or other hairy areas with partial or complete hair loss resulting in negative psychological impact if left untreated [1, 2].

Alopecia areata is a multifactorial disease with complex pathogenesis in which autoimmune, genetic, and environmental factors interacts, leading to local interferon-γ discharge which collapses the hair follicle immune privilege (HFIP) and triggers autoreactive CD8 + T cells to attack anagen hair follicles (HF) [3]. Recently, follicular proteoglycans have been suggested to regulate the hair follicles’ cycle and growth by supporting the dermal papilla fibroblasts to induce the anagen phase and contributing to HFIP [4]. The knowledge of proteoglycan’s role in AA patients may open the way for developing proteoglycan-based therapy.

Decorin is one of the proteoglycans (PG) in the perifollicular extracellular matrix (ECM), which regulates the survival of hair follicle stem cells (HFSCs) and serves as an anagen inducer [5, 6]. Disturbed decorin expression was reported in several hair disorders [4]; however, its expression in AA has not been previously investigated.

Although AA is a clinical diagnosis, trichoscopy is useful for evaluating disease progression and treatment response [7]. Trichoscopic features of AA include yellow dots (YD), black dots (BD), exclamation mark hair (EMH), and broken hairs (BH) [8].

Several treatment options are available; however, there is no uniformly effective medication for this disease [9]. The most popular treatments are corticosteroids, both topical and intralesional [10]. Also, several studies documented the usefulness of platelet-rich plasma (PRP) [11–13], vitamin D analogs [14], and fractional lasers (FCL) in AA [15–17]. However, to the best of our knowledge, no previous studies compared the effect of FCL alone versus its combination with Triamcinolone acetonide (TA), PRP, or vitamin D in treating AA.

This study aims to compare the direct effect of FCL as a monotherapy for AA versus its impact on assisted drug delivery of (TA, PRP, or vitamin D). Then, assess the histopathologic features and immunohistochemical expression of Decorin in AA lesions before and after treatment.

## Patients and methods

This randomized clinical trial (ClinicalTrials.gov ID: NCT04003376) was conducted at (Dermatology, Venreology and Andrology, and Pathology Departments) Assiut University. From September 2020 to February 2022. The study was approved by the Institutional Ethical Review Board of the Faculty of Medicine, Assiut University (IRB: 17200357).

### Patients selection

Patients with patchy AA of any hairy area and those with ophiasis AA, ≥ 18-year-old, of both sexes, who did not receive treatment for AA in the past month before joining the study were included. Pregnant and lactating females and patients with alopecia totalis/alopecia universalis, active infections, hypertrophic scars/keloids, hypercalcemia, renal/liver/blood disorders, and those using antiplatelet therapy were excluded.

**Fig. 1:**
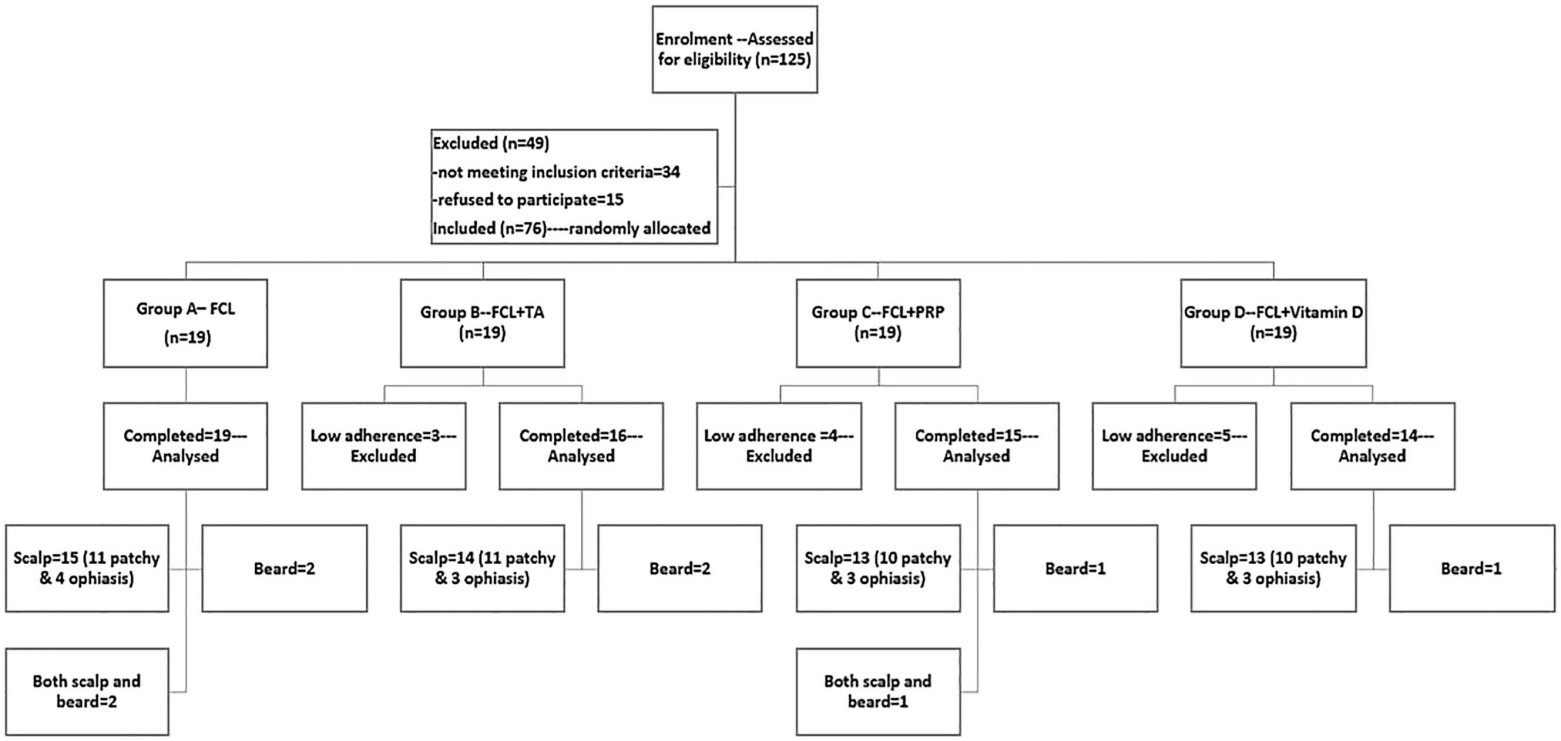
Flow chart for the study patients. *FCL* Fractional Carbon dioxide Laser, *TA* Triamcinolone Acetonide, *PRP* Platelet-Rich Plasma

After obtaining informed consent from the participants, patients were randomly assigned (using computer-based randomization) into four treatment groups, as shown in the flow chart, Fig. 1.

History was obtained, including age, disease onset and duration, previous treatment, past/family history of AA, and any associated medical problems. Clinical examination for AA sites, nail involvement, and laboratory investigations: complete blood picture, kidney/liver functions, and serum calcium were done.

### Technique

First, cleaning the lesions with ethyl alcohol 70% and application of Lidocaine 5% gel (30 minutes) was done, then all patients received monthly FCL (Multixel, Daeshin enterprise co., Korea) sessions for a maximum of six sessions; using pulse energy:30.7–40.3 mj [15], 100 spots/cm^2^ and depth 1 for 2 passes.

And according to the patient’s treatment group, the following was done:

- **Group A:** FCL was used as the sole intervention.
- **Group B**: FCL was followed by topical application of TA solution (Epirelefan vials 40mg/ ml, Epico, Egypt), dilution 5 mg/ml, 0.1 ml/cm^2^, maximum 3 mL/session [18].
- **Group C:** FCL was followed by topical application PRP. PRP was prepared by centrifugation of 10 ml of blood withdrawn on 1 ml of sodium citrate at 160 g, then 400 g, 10 minutes each [19].
- **Group D:** Patient FCL was followed by topical application of vitamin D3 solution (Devarol^®^ ampoule 200,000 IU/2 ml, Memphis, Egypt), the maximum amount applied/session: 1 ml, i.e., 100,000 IU [20].

### Patients’ evaluation

Patients were evaluated at the baseline, before each session, and 3 months after the last treatment using:

- Alopecia areata severity index (AASI). The percentages of change in AASI = $\frac{(\mathrm{pretreatment AASI}-\mathrm{post}-\mathrm{treatment AASI})}{ \mathrm{pretreatment AASI}}\times 100.$ Then the result was graded as: 0 to < 25%—no improvement, 25 to < 50%—mild improvement, 50 to < 75%—moderate improvement, and 75 to 100% —excellent improvement [17].
- MacDonald Hull and Norris grading system to evaluate the response in each lesion separately [21]: Grade 0—no hair re-growth, Grade 1—vellus hair re-growth, Grade 2—few pigmented terminal hair re-growth, Grade 3—clusters of terminal hair and Grade 4—full terminal hair re-growth, then the mean grade for lesions in each group was calculated.
- Photography was done using Canon EOS 4000D camera (18 Megapixels, Canon Inc., Taiwan).
- Trichoscopic examination was done using Dermlite DL4 (3Gen, San Juan Capistrano, CA, USA)
- Patients were asked to report adverse events experienced during/after the treatment sessions.

Pain during the procedure was assessed each session using a numeric pain rating scale (NRS-10), which ranges from 0 to 10; the mean value for each patient in all sessions was calculated. The score was interpreted as • 0—no pain • 1–3—mild pain • 4–6—moderate pain • 7–10—severe pain [22].

### Tissue specimens

Obtaining informed consent from those who agreed, punch biopsies (4 mm) were obtained from the periphery of AA scalp lesions of 46 patients: 13, 11, 10, and 12 patients in groups A, B, C, and D respectively. Two biopsies were obtained from each patient, one at the baseline and the other one month after the last session. Additionally, 13 biopsies from the scalp of age and sex-matched healthy volunteers were obtained as controls.

All biopsies were formalin-fixed for 24 h, cut according to the HoVert technique, and prepared for routine histopathologic examination.

The histological changes in the lesional AA scalp specimens before and after treatment were reported and compared to healthy skin. First, the follicles were classified based on the hair shaft diameter into vellus and terminal. Then, terminal follicles were classified based on the phase of the hair cycle into anagen, catagen, and telogen [23]. Telogen and catagen follicles were grouped together as they represent stages in the continuum [24–26].

The following were counted: the number of terminal and vellus hairs, terminal anagen and terminal telogen/catagen HF, and the total number of follicles which is the sum of (terminal anagen, terminal telogen/catagen, and vellus hairs).

### Immunohistochemical staining

Four μm-thick sections were performed from formalin-fixed paraffin-blocks, followed by deparaffinization and rehydration. Hydrogen peroxide 3% was used to block the endogenous peroxidase. Immersion in 10 mmol/l citrate buffer (pH 6.0) in a microwave at 90 °C for 15 min was then performed for antigen retrieval. Then, primary mouse monoclonal antibody against Decorin (9XX) of human origin (sc-73896, Santa Cruz Biotechnology, CA, USA) at dilution of 1:100 was added, and the slides were incubated at room temperature for 1 h. Secondary staining kits were applied according to the manufacturer's instructions (ScyTek Laboratories, Logan, UT, USA). Cytoplasmic expression of Decorin was considered positive, and its immunohistological signal intensity was semiquantitatively assessed (graded as 0, negative; 1, weak; 2, moderate; 3, strong) [27].

### Statistical analysis

Analysis was done using the Statistical Package for Social Science version 22. Data were presented as percentage, mean, standard deviation, median, and interquartile range. Tests used: chi-square (*χ*^2^) for categorical variables; Mann–Whitney and Kruskal–Wallis for quantitative variables between two groups. McNemar’s and Wilcoxon signed-rank tests compared quantitative variables pretreatment versus post-treatment. Spearman correlation measured correlations between quantitative variables. *P* value was considered significant when ≤ 0.05.

## Results

Sixty-four patients completed the study, 19 patients in group A, 16 in group B, 15 in group C, and 14 in group D. There was no significant difference regarding our patients’ demographic and clinical data, as shown in Table 1.

**Table 1:** Demographics and clincal data of the studied groups

	Group A *n* = 19	Group B *n* = 16	Group C *n* = 15	Group D *n* = 14	Total *n* = 64	*p* value
Sex	*M* = 10(52%) *F* = 9(48%)	*M* = 8(50%) *F* = 8 (50%)	*M* = 8(53%) *F* = 7(47%)	*M* = 8(57%) *F* = 6(43%)	*M* = 34(53%) *F* = 30(47%)	0.9^a^
Age (years) Mean ± SD	27.2 ± 9.2	27 ± 10.9	27 ± 7.1	25.2 ± 8.8	26.7 ± 8.9	0.9^b^
*Duration of the current episode of AA (month)*						
Median (IQR) Mean ± SD	4(1–8.5) 17 ± 33	8(3–18) 15 ± 20	9(3–48) 25 ± 28	6.5(3.3–14.3) 14 ± 22	6(2.75–18) 18.3 ± 26.7	0.65^b^
*Site of hair loss*						
Scalp	15(79%)	14(87.5%)	13(87%)	13(93%)	55(85.9%)	0.9 ^a^
Scalp and beard	2(10.5%)	0	1(6.5%)	0	3(4.6%)	0.8 ^a^
Beard alone	2(10.5%)	2(12.5%)	1(6.5%)	1(7%)	6(9.3%)	0.9 ^a^
*Pattern of hair loss*						
Ophiasis	4(21%)	3(18.75%)	3(20%)	3(21.4%)	13(20.3%)	0.99 ^a^
Patchy	15(79%)	13(81.25%)	12(80%)	11(78.6%)	51(79.6%)	
Number of lesions in each group	40	62	45	38	185	0.684^a^
Nail affection	2(10.5%)	3(18.75%)	1(6.5%)	3(21.4%)	9(14%)	
Past history of AA	7(36.8%)	11(68.75%)	9(60%)	4(28.6%)	31(48.4%)	
Family history	8(42.1%)	6(37.5%)	10(66.67%)	2(14.3%)	26(40.6%)	

Note: Chi-square test^a^; Kruskal–Wallis test^b^. M: Male, F: Female.

### Clinical evaluation of the response

**Fig. 2:**
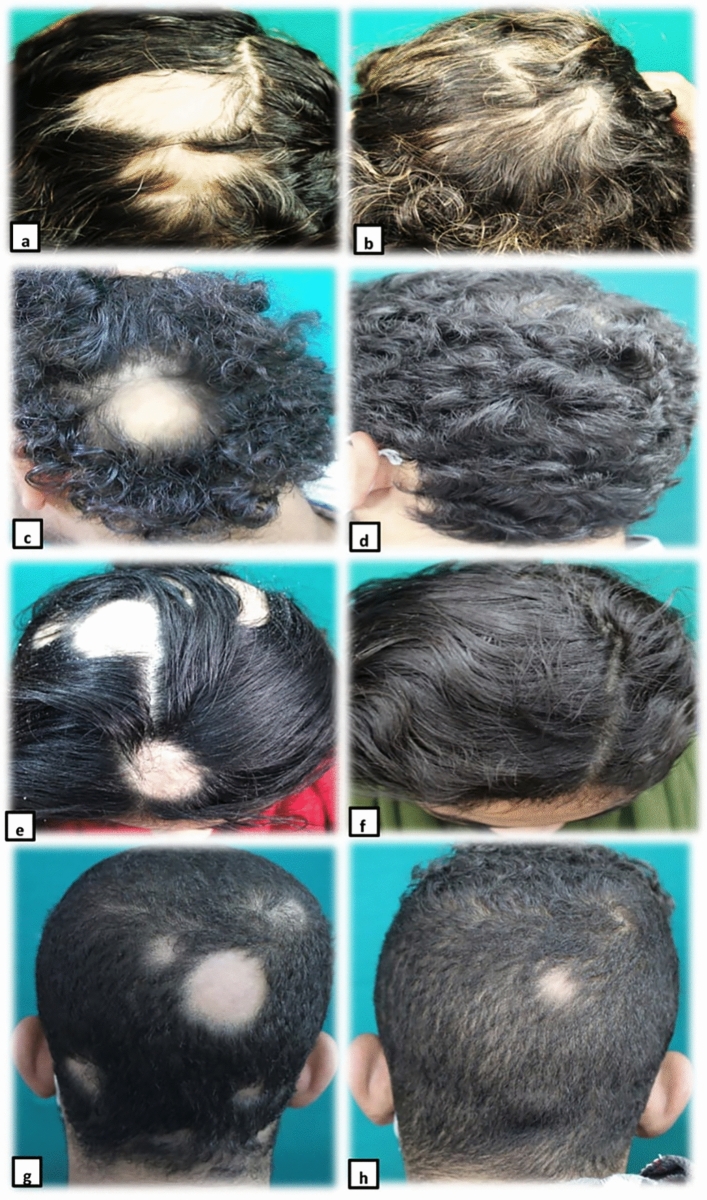
Excellent improvement in a female patient with patchy alopecia areata in group A, (**a**: before treatment, **b**: after treatment); Excellent improvement in a male patient with patchy alopecia areata in group B (**c**: before treatment, **d**: after treatment); Excellent improvement in a female patient with patchy alopecia areata in group C (**e**: before treatment, **f**: after treatment); Moderate improvement in a male patient with patchy alopecia areata in group D (**g**: before treatment, **h**: after treatment)

**Fig. 3:**
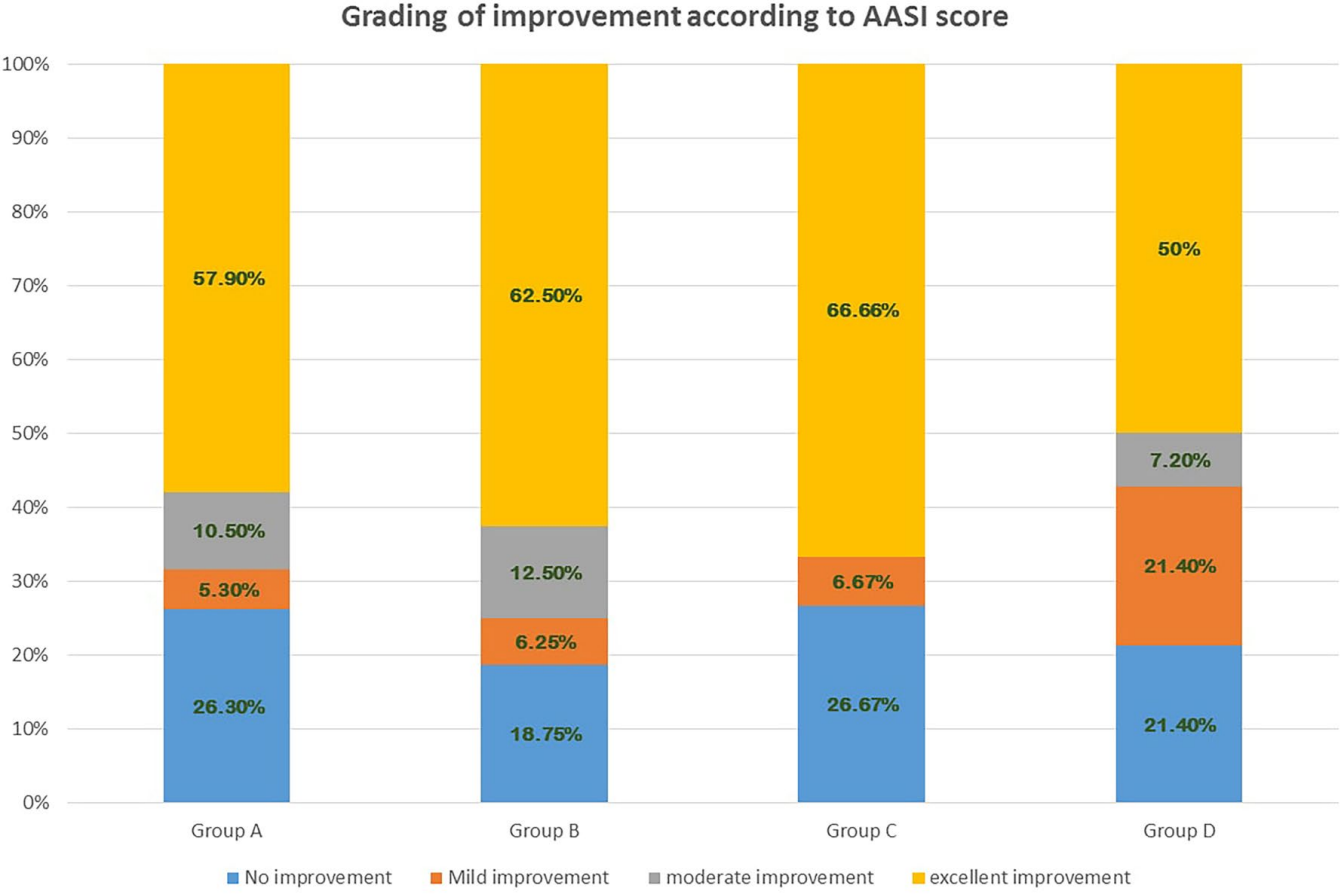
Grading of patients improvement according to Alopecia areata severity index

All treated groups showed significantly decreased AASI as compared to the baseline (*p* < 0.05). The best response was observed in group B (72.6%), and the least percentage of improvement (57.5%) was in group A with no significant difference between the four groups (Table 2, Fig. 2). Grading the patients’ improvement according to AASI is shown in Fig. 3.

**Table 2:** Alopecia areata severity index (AASI) before & after treatment among the four studied groups

AASI (patients with scalp &/or beard AA)	Group A *n* = 19	Group B *n* = 16	Group C *n* = 15	Group D *n* = 14	Total *n* = 64	*p*1^a^	*p2*^a^	*p3*^a^	*p4*^a^	*p5*^a^	*p6*^a^	*p7*^c^
*Baseline AASI*												
Mean ± SD Median	11.3 ± 12.9 9	12.9 ± 9.9 9.5	16 ± 14.8 9	12.5 ± 9.8 8.5	12.7 ± 11.7 9	*0.7*	*0.2*	*0.3*	*0.5*	*0.7*	*0.9*	*0.9*
*Post-treatment AASI*												
Mean ± SD Median	5.7 ± 8 2.5	4.8 ± 7.7 0.4	6.4 ± 9.9 0.6	7.4 ± 10.5 2.6	6 ± 8.8 1.3	0.3	0.2	0.2	0.2	0.2	0.9	0.7
*Change in AASI*												
Mean ± SD Median	5.2 ± 7.4 2	7.3 ± 7.1 4.7	9.6 ± 12.6 4.2	5 ± 5.1 4	6.7 ± 8.5 3.1							
*Percentage of improvement AASI*												
Mean ± SD	57.5 ± 42	72.6 ± 36.5	65.8 ± 39.9	61.7 ± 41	64.1 ± 39.5	0.9	0.9	0.9	0.9	0.9	0.9	0.9
*p* value ∞^b^	0.01*^b^	0.006*^b^	0.02*^b^	0.02* ^b^	0.002*^b^							

*n* = number of patients in each group, Mann–Whitney test^a^; Wilcoxon-sign test^b^, Kruskal–Wallis test^c^, *p* value ∞ (baseline & post-treatment AASI), *p1*(A,B), *p2*(A,C), *p3*(A,D), *p4*(B,C), *p*5(B,D), *p*6(C,D), *p*7(A,B,C,D). AASI: Alopecia Areata Severity Index

*Statistical significance

MacDonald Hull and Norris grading for evaluation of each lesion showed a significant post-treatment increase in the mean grade of the lesions in all groups compared to the baseline (Table 3).

**Table 3:** MacDonald Hull and Norris grading for individual lesions among patients of different groups before and after treatment

MacDonald Hull and Norris grading	Group A *N*` = 40	Group B *N*` = 62	Group C *N*` = 45	Group D *N*` = 38	Total *N*` = 185	*p*1^a^	*p2*^a^	*p3*^a^	*p4*^a^	*p5*^a^	*p6*^a^	*p7*^c^
*Baseline*												
G0	18 (45%)	39(62.9%)	30(66.6%)	17(44.7%)	104(56.2%)							
G1	12 (30%)	15(24.2)	7(15.6%)	9(23.7%)	43(23.3%)							
G2	10 (25%)	7(11.3%)	7(15.6%)	8(21.1%)	32(17.3%)							
G3	0 (0%)	1 (1.6%)	1(2.2%)	4(10.5%)	6(3.2%)							
G4	0 (0%)	0(0%)	0(0%)	0(0%)	0(0%)							
Mean SD	0.8 0.82	0.52 0.76	0.53 0.84	0.97 1.05	0.67 0.87	0.09	0.1	0.1	0.3	0.2	0.1	0.09
*After treatment*												
G0	2 (5%)	5 (8.1%)	1(2.2%)	2(5.3%)	11(5.9%)							
G1	5(12.5%)	4 (6.4%)	4(8.9%)	4(10.5%)	15(8.1%)							
G2	2(5%)	5 (8.1%)	6(13.3%)	4(10.5%)	14(7.6%)							
G3	10(25%)	9 (14.5%)	9(20%)	3(7.9%)	32(17.3%)							
G4	21(52.5%)	39(62.9%)	25(55.6%)	25(65.8%)	113(61.1%)							
Mean SD	3.0.08 1.24	3.18 1.3	3.18 1.11	3.16 1.29	3.16 1.23	0.4	0.7	0.4	0.5	0.8	0.6	0.7
*p* value ∞	0.00001* ^b^	0.00001* ^b^	0.00001* ^b^	0.00001* ^b^	0.00001* ^b^							

*N*` = number of lesions in each group. Mann–Whitney test^a^, Wilcoxon test ^b^; Kruskal–Wallis test^c^, *p* value ∞ (baseline & post-treatment MacDonald Hull and Norris grade), *p1*(A,B), *p2*(A,C), *p3*(A,D), *p4*(B,C), *p*5(B,D), *p*6(C,D), *p*7(A,B,C,D)

*Statistical significance

### Trichoscopic evaluation

**Fig. 4:**
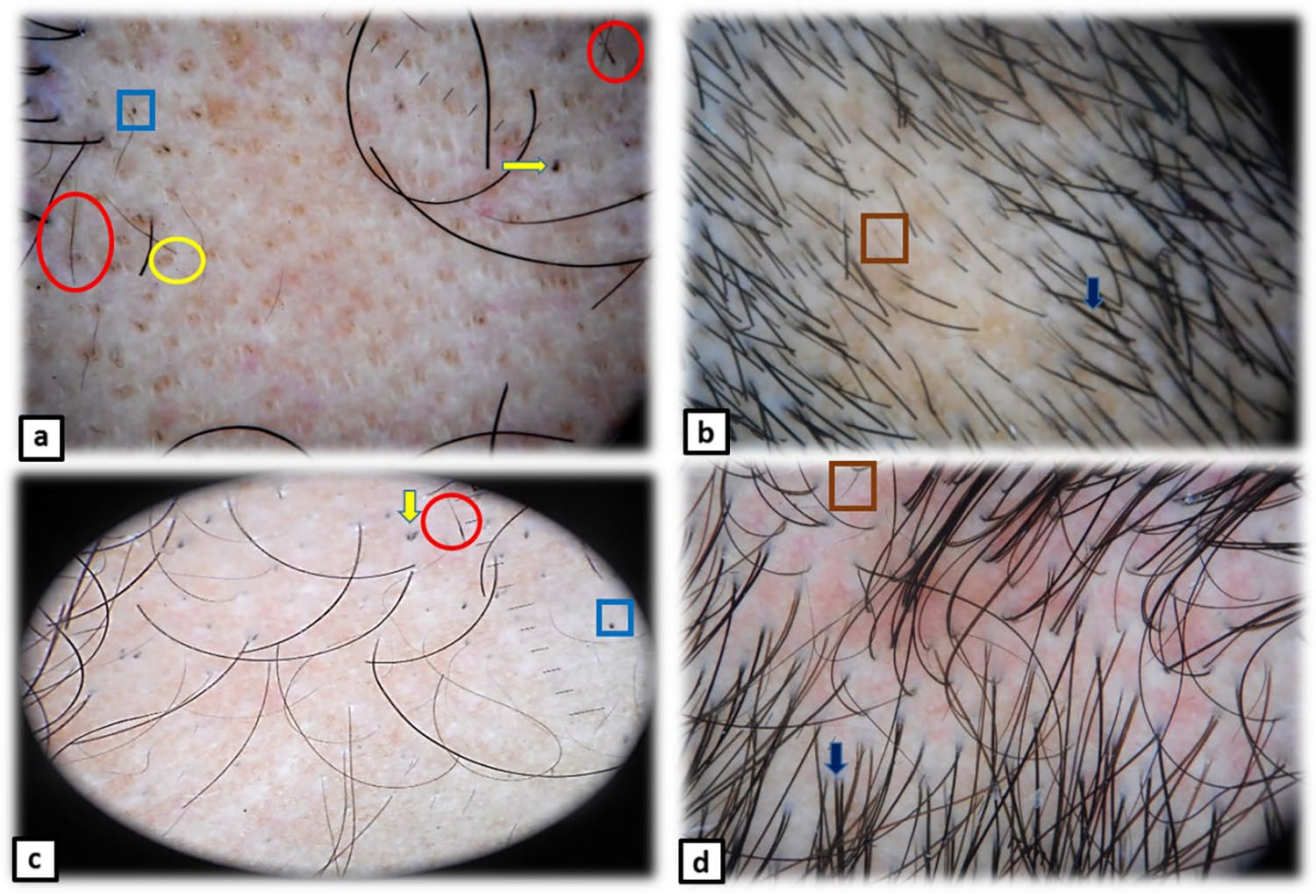
Trichoscopic images of alopecia areata patients. **a** and **c** Before treatment, showing exclamation mark hair—red circles, black dots—blue squares, broken hairs—yellow arrows, yellow dots—yellow circles. **b** and **d** After treatment, showing disappearance of all features of AA activity and appearance of terminal hair—blue arrows and short vellus hair—brown squares

Compared to the baseline, all treatment groups showed significant post-treatment reduction in BD, BH, EMH, and YD and increase in terminal hair with no significant difference between the four groups (Table 4). Trichoscopic features of AA before and after treatment are shown in Fig. 4.

**Table 4:** Trichoscopic features’ frequencies among patients of the four groups before and after treatment

Trichoscopic features	Group A *n* = 19	Group B *n* = 16	Group C *n* = 15	Group D *n* = 14	Total	*P*1^*b*^	*P*2^*b*^	*P*3^*b*^	*P*4^*b*^	*P*5^*b*^	*P*6^*b*^	*P7*^c^
YD 1	11 (57.9%)	10 (62.5%)	9(60%)	9(64.3%)	39(60.9%)	0.9	0.6	0.9	0.9	0.9	0.9	0.9
YD 2	2 (10.5%)	3 (6.25%)	2 (13.33%)	2(14.3%)	9(14%)	0.9	0.9	0.9	0.9	0.9	0.9	0.9
*p* value∞ ^a^	0.004*	0.01*	0.01*	0.01*	0.001*							
BD 1	10 (52.6%)	8 (50%)	8 (53.3%)	9(64.3%)	35(54.6%)	0.9	0.9	0.9	0.9	0.9	0.9	0.8
BD 2	1 (5.2%)	0	0 (0%)	1 (7.1%)	2(3.1%)	0.7	0.1	0.7	0.7	0.8	0.7	0.5
*p* value∞ ^a^	0.004*	0.004*	0.008*	0.008*	0.001*							
BH 1	9 (47.4%)	7 (43.75%)	7 (46.67%)	6 (42.9%)	29(45.3%)	0.9	0.6	0.9	0.9	0.9	0.9	0.9
BH 2	0	0	0	0	0							
*p* value∞ ^a^	0.004*	0.01*	0.01*	0.03*	0.001*							
EMH 1	6 (31.6%)	9 (56.25%)	10(66.67%)	8(57.1%)	33(51.5%)	0.9	0.9	0.9	0.9	0.9	0.9	0.1
EMH 2	0	1 (6.25%)	3 (20%)	2 (14.3%)	6(9.3%)	0.7	0.1	0.7	0.7	0.8	0.7	0.2
*p* value∞ ^a^	0.03*	0.008*	0.01*	0.03*	0.001*							
SVH 1	9(47.4%)	10 (62.5%)	7 (46.67%)	8 (57.1%)	34(53.1%)	0.9	0.6	0.9	0.9	0.9	0.9	0.7
SVH 2	10(52.6%)	12 (75%)	8 (53.3%)	10(71.4%)	40(62.5%)	0.9	0.7	0.6	0.9	0.9	0.9	0.2
*p* value∞ ^a^	0.9	0.6	0.9	0.7	0.3							
Terminal hair 1	3	1	1	1	6	0.9	0.6	0.9	0.9	0.9	0.9	0.2
Terminal hair 2	14(73.7%)	13(81.25%)	12(80%)	12(85.7%)	51(79.6%)	0.9	0.6	0.9	0.9	0.9	0.9	0.9
*p* value ∞ ^a^	0.001*	0.001*	0.001*	0.001*	0.0001*							

1: Before treatment, 2: After treatment, *YD* yellow dots, *BD* black dots, *BH* broken hairs, *EMH* exclamation mark hairs, *SVH* short vellus hair; Mc-Nemartest^a^; Chi-square test^b^, Kruskal–Wallis test^c^*, p value* ∞ (before &after treatment). *P*1(A&B), *P2(A&C), P3(A&D), P4(B&C), P5(B&D), P6(C&D), P7,*(A,B,C &D)

*Statistical significance

### Safety assessment

All patients reported post-treatment erythema and scaling. Only 10 cases (15.6%) reported mild post-treatment headaches, resolved spontaneously within 24 h. Mild to moderate pain during the procedure was reported. The mean NRS = 2.8 ± 1.7.

### Recurrence

In the 3-month follow-up duration, only one patient (in group D) developed recurrence.

### Correlations

Significant negative correlations existed between the percentage of improvement in AASI and the patient’s age (*r* = − 0.3, *p* = 0.01) and disease duration (*r* = − 0.4, *p* = 0.0001).

### Histopathological and immunohistochemical results

Histologic examination of scalp biopsies of the controls revealed uniformly distributed follicular units. The mean number of HF was 22.7 ± 2.9, with increased number of anagen follicles 18.9 ± 2.3. The mean number of terminal and vellus follicles was 20.9 ± 2.1 and 2.9 ± 1.5, respectively.

In all AA groups before treatment, the mean total number of follicles and anagen hair was significantly decreased, while the mean number of Telogen/catagen follicles and vellus hair was significantly increased compared to the control group. Fibrous stelae and peribulbar lymphocytic infiltrate were detected in 57% and 28.6% of the cases, respectively.

**Fig. 5:**
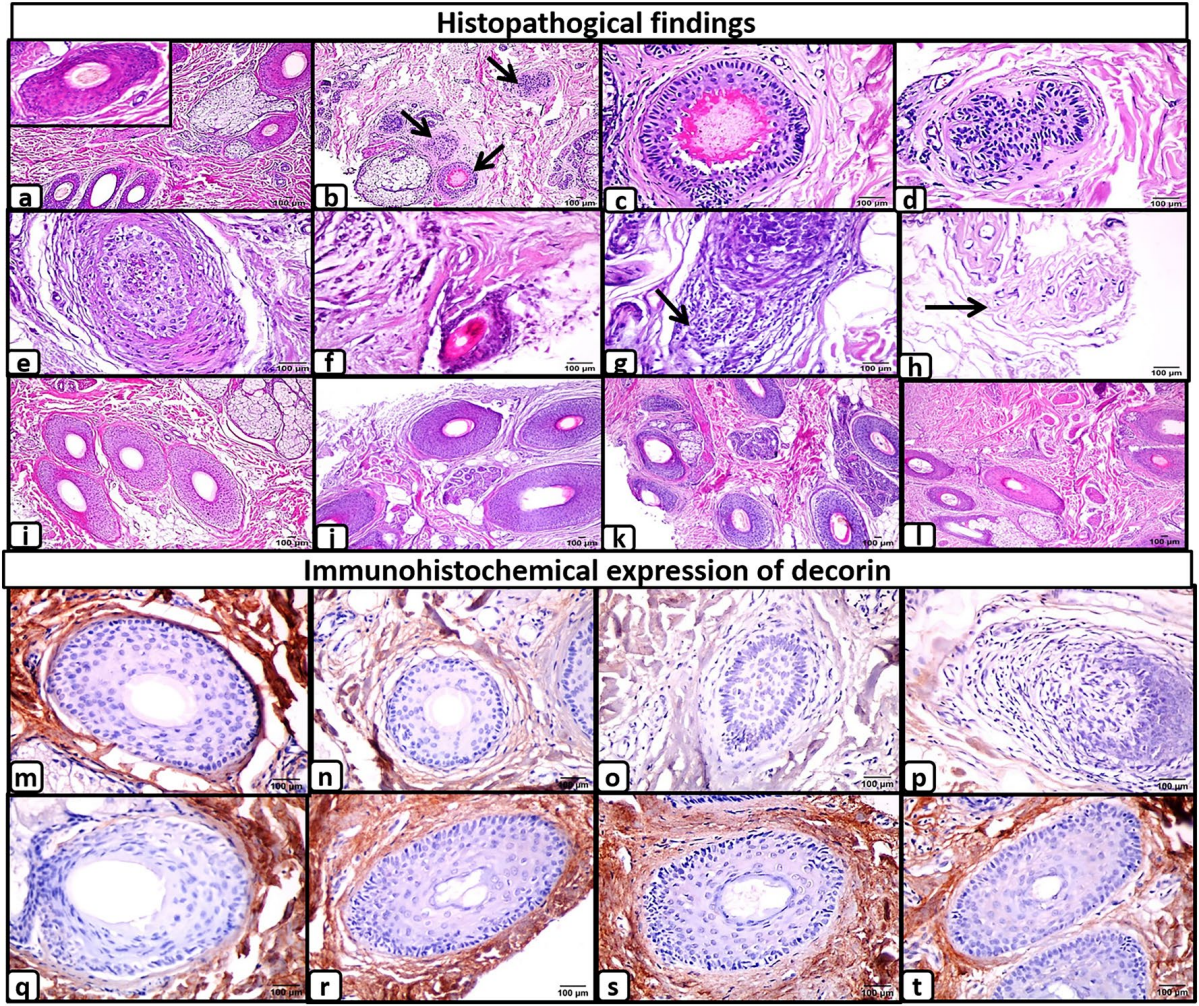
Histopathologic examination of the scalp biopsies of the study groups (scale bar 100 µm): **a** Uniformly distributed follicular units with multiple anagen follicles (inset) in the control group (× 200). **b** Increased number of the telogen hairs (arrows) in AA before treatment (× 200). Higher magnification (× 400) of scalp biopsies from AA group before treatment shows: **c** telogen hair, **d** telogen germinal unit, **e** catagen hair, **f** vellus hair, **g** Peribulbar lymphocytic inflammation and **h** fibrous stelae. (**i**–**l**, × 200) Increased number of anagen hair follicles in the treated groups. Immunohistochemical expression of decorin, **m** Strong expression of decorin in the peri-follicular extracellular matrix (ECM) of the anagen hair follicles in the control group (× 400). **n** Weak decorin expression in the perifollicular ECM of the anagen hair follicles in AA group before treatment (× 400). **o** and **p** loss of decorin expression in the perfollicular ECM of the telogen hair and in the areas of perifollicular inflammation (× 400) respectively. **q**–**t** Increased decorin expression in the perifollicular ECM of the anagen hair follicles in treated groups

Histologic improvement was observed after treatment in all groups, with increased average number of anagen follicles and decreased average number of Telogen/catagen follicles compared to the baseline. The pathologic features of AA before and after treatment compared to the control scalp are summarized in Table 5 and Fig. 5.

**Table 5:** Pathological features of alopecia areata and immunohistochemical expression of Decorin before and after treatment in comparison with healthy controls

	Group A *n* = 13	Group B *n* = 11	Group C *n* = 10	Group D *n* = 12	Control *n* = 13	*P*1^b^	*P*2^*b*^	*P*3^b^	*P*4^b^	*P*5^b^	*P6*^b^	*P*7^b^	*P8* ^b^	*P9*^b^	*P10*^b^
*Histopathological features: Follicles count*															
Total follicles 1 count	13 ± 1.7	13.9 ± 1.64	14.6 ± 1.9	14.2 ± 1.8	22.7 ± 2.9	0.9	0.9	0.9	0.9	0.9	0.9	0.0001*	0.0004*	0.008*	0.007*
Total follicles 2 count	16.7 ± 3.9	19 ± 3	19.7 ± 2.6	17 ± 4.4	22.7 ± 2.9	0.9	0.9	0.9	0.9	0.9	0.9	0.002*	0.3	0.9	0.01*
*p* value∞^a^	0.07	0.01*	0.04*	0.2	0.9										
Anagen follicles 1	2.7 ± 2.1	2.5 ± 1.8	2.9 ± 2.2	2.9 ± 2.3	18.9 ± 2.3	0.9	0.9	0.9	0.9	0.9	0.9	0.0001*	0.0002*	0.0008*	0.0004*
Anagen follicles 2	10.6 ± 6.1	14.6 ± 8	14.3 ± 7.4	12.6 ± 8.9	18.9 ± 2.3	0.9	0.9	0.9	0.9	0.9	0.9	0.03*	0.07	0.9	0.5
*p* value∞^a^	0.05	0.01*	0.01*	0.01*	0.9										
Telogen/catagen follicles 1	7.1 ± 2.4	6.6 ± 2.4	7.1 ± 2.6	6.9 ± 2.5	1.9 ± 1	0.9	0.6	0.9	0.9	0.9	0.9	0.0002*	0.0001*	0.0006*	0.0002*
Telogen/Catagen follicles 2	2.4 ± 2.4	1.3 ± 1.8	2 ± 2.4	1.1 ± 2.3	1.9 ± 1	0.9	0.9	0.4	0.9	0.9	0.9	0.9	0.9	0.9	0.3
*p* value∞^a^	0.04*	0.05	0.01*	0.03*	0.9										
Terminal hair 1	9.8 ± 4.3	9.1 ± 3.7	10 ± 4.6	9.8 ± 4.4	20.9 ± 2.1	0.9	0.9	0.9	0.9	0.9	0.9	0.0002*	0.0002*	0.0006*	0.0003*
Terminal hair 2	13 ± 0.4.9	16 ± 6.8	16.3 ± 5.7	13.7 ± 7.6	20.9 ± 2.1	0.9	0.9	0.9	0.9	0.9	0.9	0.006*	0.5	0.7	0.059
*p* value∞^a^	0.03*	0.008*	0.01*	0.03*	0.9										
Vellus hair1	4.6 ± 3.9	4.7 ± 3.2	4.6 ± 4.1	4.4 ± 3.3	2.9 ± 1.5	0.9	0.6	0.9	0.9	0.9	0.9	0.0002*	0.0002*	0.0007*	0.0001*
Vellus hair2	3.8 ± 5.1	3.1 ± 1.6	3.8 ± 2.9	3.8 ± 3.4	2.9 ± 1.5	0.9	0.9	0.9	0.9	0.9	0.9	0.9	0.9	0.9	0.9
*p* value∞^a^	0.9	0.6	0.9	0.7	0.3										
*Intensity of immunohistochemical expression of decorin before treatment N (%)*															
Negative = 0	2(15.4%)	1(9%)	0(0%)	1(8.3%)	0(0%)										
Weak = 1	11(84.6%)	10(91%)	10(100%)	11(91.6%)	1(7.7%)										
Moderate = 2	0(0%)	0(0%)	0(0%)	0(0%)	2(15.4%)										
Strong = 3	0(0%)	0(0%)	0(0%)	0(0%)	10(76.9%)										
Mean intensity ± SD	0.84 ± 0.37	0.8 ± 0.4	1 ± 0	0.81 ± 0.38	2.7 ± 0.63	0.9	0.8	0.9	0.7	0.9	0.8	0.0001*	0.0001*	0.0001*	0.0001*
*Intensity of immunohistochemical expression of decorin after treatment* *N* (%)															
Negative = 0	0	0	0	0	0										
Weak = 1	3(23%)	3(27%)	3(30%)	4(33%)	1(7.7%)										
Moderate = 2	6(46%)	2 (18%)	2 (20%)	3(25%)	2(15.4%)										
Strong = 3	4 (31%)	6 (55%)	5 (50%)	5(42%)	10(76.9%)										
Mean intensity ± SD	2.1 ± 0.75	2.3 ± 0.9	2.2 ± 0.9	2.1 ± 0.9	2.7 ± 0.63	0.9	0.9	0.9	0.3	0.9	0.9	0.7	0.9	0.6	0.3
p value∞^a^	0.01*	0.01*	0.01*	0.01*	0.9										

1 = before treatment, 2 = after treatment. N = number of cases with specific intensity in each group, *p value* ∞ (before &after treatment).*P*1(A&B), *P2(A&C), P3(A&D), P4(B&C), P5(B&D), P6(C&D), P7, P8, P9, P10 (healthy controls &groups A,B,C&D respectively).*Wilcoxon test ^a^, Mann–Whitney test ^b^, * = Statistical significance.

Regarding immunohistochemical detection of Decorin, in the control group, Decorin was expressed in the entire dermis, co-distributing with collagen fibers, while it was negative in the epidermis and appendages. Additionally, strong expression was detected in the ECM of anagen follicles in 10 (76.9%) specimens.

In AA groups before treatment, decorin expression was markedly decreased in the ECM of anagen follicles. It was lost in the ECM surrounding telogen/catagen follicles and areas of perifollicular inflammation. The mean decorin expression was significantly lower in all groups (0.84 ± 0.37, 0.8 ± 0.4, 1 ± 0, 0.81 ± 0.38 for groups A, B, C, and D, respectively) as compared to the control group (2.7 ± 0.36) (*p* = 0.0001). After treatment, there was a significant increase in decorin expression in all treated groups, mainly around anagen follicles, compared to the baseline specimens that approached the control level (Table 5, Fig. 5).

## Discussion

Alopecia areata is a complex autoimmune hair loss disorder [28]. Although many treatment options exist, most treatments have limited efficacy and/or serious adverse effects [29]. This encourages the ongoing evolution of new treatments [30].

This research was the first to compare the efficacy of FCL monotherapy for AA versus its combination with other treatments (TA, PRP, and vitamin D3 solution).

Fractional laser is a novel unconventional therapy for AA [31]. That may induce hair re-growth in AA via triggering T cell apoptosis [32, 33], stimulating Wnt/β catenin pathways in the HF [34, 35]. Moreover, fractional ablative lasers create wounds that activates HFSCs [36], and generate microscopic channels of skin ablation that provide access to topically applied drugs [37].

In group A, patients received FCL sessions only, and there was significant improvement in AASI at the end of the study compared to the baseline. The mean percentage of improvement was 57.5 ± 42%, with 57.9% of the patients showing ≥ 75% improvement.

These results agreed with El Husseiny et al., who described a significant improvement in the patches treated with FCL, with 60% of their patients showed > 75% improvement [15].

Nouh et al. reported significant increase in the re-growth scale of 40 AA patients treated with FCL alone; excellent response (≥ 75%) was observed in 15% of their patients. Our superior results may be explained by the lower number of laser sessions (3 sessions) and the different laser parameters used by Nouh et al. [38].

On the contrary, Yalici-Armagan and Elcin reported that FCL was unsuccessful in treating AA [39]. They only included patients with long disease duration (≥ 12 months), which may explain their poorer outcomes.

Topical and Intralesional corticosteroids (ILC) are the first-line treatments for limited AA [40]. In adults with patchy disease, ILC is preferred over topical steroids for their faster, longer action, and deeper penetration [41]. However, ILCs are associated with intolerable pain. Laser-assisted drug delivery (LAD) overcomes this obstacle and allows uniform, deeper penetration of topical agents with much less pain [17, 42].

Our group B patients who were treated by FCL + TA showed significant decrease in the post-treatment AASI compared to the baseline. The mean percentage of improvement was 72.6 ± 36.5. Excellent improvement (≥ 75%) was achieved by 62.5%.

In agreement with us, Soror et al. reported significant improvement in grading AA patches treated by FCL plus TA [42]. Also, Abd El Kawy et al. observed ≥ 75% improvement (according to severity of alopecia tool) in 56.7% of AA patches treated by FCL + TA [43].

Majid et al. reported excellent response in 87.5% of AA patients treated by FCL combined with TA. Their better results may be related to the smaller sample size (8 patients) and the use of higher concentrations of TA (10 mg/ml), and different laser parameters [16].

Also, Issa et al. also treated 3 AA patients using FCL and TA followed by acoustic pressure wave ultrasound. After one session, two patients had excellent responses [44].

The additive use of ultrasound waves, the higher TA concentration (20 mg/ml), the smaller sample size, and the different laser protocol applied can explain their better results.

Platelet-rich plasma (PRP) is widely used to treat hair loss disorders, e.g., AA and Androgenetic alopecia [45]. Growth factors released from platelets’ α-granules enhance blood flow around the HF, stimulate cellular proliferation, prolong anagen and inhibit inflammation [46].

In this study, group C patients were treated using FCL followed by PRP topical application. Excellent improvement was observed in ten patients (66.6%), according to AASI.

Ragab et al. reported excellent improvement in 40% and moderate improvement in 20% of their group of patients treated by FCL plus PRP for three sessions [17].

Our superior results may be related to our study’s greater number of treatment sessions, the different PRP preparation protocols, and laser parameters used.

The association of AA with vitamin D deficiency is well documented. Vitamin D's powerful anti-inflammatory and immunoregulatory actions justified its use in treating AA [47, 48].

Recently, Rashad et al. described the efficacy of intralesional injection of vitamin D3 solution in AA [41]. However, to the best of our knowledge, our study is the first in which LAD of Vitamin D3 solution is employed for treating AA.

In this study, we treated group D patients using FCL followed by topical application of Vitamin D3 solution. This yielded significant decrease in the post-treatment AASI compared to baseline. The mean improvement percentage was 61.7 ± 41. Improvement of ≥ 75% was observed in 50% of the patients.

Similarly, Rashad et al. treated 30 AA patients with intra-dermal injections of vitamin D3 monthly for 3 months. They reported ˃ 75% hair re-growth in 50% of their patients [41].

Trichoscopy is a noninvasive instrument that differentiates progressive from stationary disease and heralds disease recovery before it can be observed clinically [49].

In our study, we noticed YD, BD, BH, EMH, and short vellus hairs as the most frequent dermoscopic features in AA, and this was in agreement with Inui [8].

All groups showed significant post-treatment decline in YD, BD, BH, EMH, parallel with significant appearance of terminal hairs in 79.6% of the patients compared to the baseline. These results agreed with those reported by other studies [14, 41].

In this study, mild to moderate pain occurred during FCL sessions, and the mean NRS for pain was 2.8 ± 1.7. Similar pain score (3.1) was reported by Ragab et al. in his group of patients treated by FCL + PRP [17]. However, Soror et al. reported lower pain score (1.67) for AA patches treated by FCL + TA [42].

All patients developed transient post-treatment erythema and scaling; similar side effects were reported by EL Husseiny et al. [15].

Ten patients developed tolerable headache after treatment that resolved without treatment in < 24 h. Ragab et al. described post-treatment mild headache as an adverse effect of treating AA using FCL + PRP [17].

In our study, at the 3-months follow-up visit, only one patient in group D experienced relapse. Studies utilizing FCL for treating AA reported no recurrence in any patient at the 3-months follow-up visit [15, 17, 38].

In agreement with previous studies [38, 43], the percentage of improvement of AASI related negatively with high statistical significance to the patient's age and disease duration.

On the contrary, El-Husseiny et al. and Rageb et al. reported no significant correlations between the therapeutic response and the patient's age or disease duration [15, 17].

Our research is unique among other studies that investigated the efficacy of FCL in treating AA, whether alone or combined with other agents, in being the first to study the histopathological features and the expression of Decorin in AA patients both before and after treatment along with comparing these findings with scalp biopsies obtained from healthy volunteers.

This research revealed that baseline biopsies from AA lesions had significantly decreased number of anagen hair follicles compared to controls. Meanwhile, telogen/catagen hair counts before treatment were significantly higher than control biopsies. Previous studies reported similar findings [26, 50].

On the other hand, post-treatment biopsies showed a significantly increased number of anagen follicles and significantly decreased number of telogen/catagen follicles in all groups compared to the baseline. Moreover, no significant differences were detected on comparing post-treatment telogen/catagen follicles number to those specimens from healthy controls.

Herz-Ruelous et al. reported a significant increase in anagen hair count and a significant decrease in the catagen/telogen follicles counts from the baseline values after treating AA lesion using UVA1 [50].

Several PG are implicated in regulating key pathways in hair growth and cycling, also disturbed PG expression and signaling were suggested in several hair diseases [4]. Decorin, an important PG in the HF mesenchyme, is crucial in initiating anagen phase, maintaining HFSCs, and stimulating the proliferation of the outer root sheath keratinocytes [5, 6].

In the current study before treatment, the mean intensity of decorin expression in AA specimens of all groups was significantly lower compared to the control group. The expression was lost in the ECM around telogen/catagen follicles. On the other hand, all groups showed significantly increased decorin expression in post-treatment specimens, mainly around anagen follicles. These findings may suggest that Decorin might play a role in AA pathogenesis.

Our results agreed with McDonagh et al., who described lost expression of chondroitin sulfate around HF in AA, chondroitin sulfate is one type of glycosaminoglycan moieties that binds core proteins to form different proteoglycans, including decorin [51].

The dynamic expression of decorin throughout the hair follicle cycle was previously reported. Decorin’s peak expression in the HF mesenchyme is at the anagen phase, and it decreases as the hair follicle entering the catagen phase [5, 52, 53].

Since AA is an autoimmune attack targeting the anagen follicles leading to their transition in the catagen and telogen phases [54], this can explain our findings of decreased decorin expression in AA lesions and the restoration of decorin expression with the increase of the anagen follicles after treatment.

Moreover, interferon-γ, produced by immune cells which is a well-known key player in the pathogenesis of AA, was found to down-regulate the expression of genes encoding Decorin [55].

Interestingly, fractional ablative lasers [56], TA [57], PRP [58], and Vitamin D [59] were all found to have a permissive effect on decorin gene expression and thus reinforce the understanding of our results in terms of the augmented post-treatment expression in Decorin.

Our study highlighted the dual role played by FCL in treating AA, both directly and synergistically with other treatments through LAD and shed lights on decorin deficiency as an important molecular change in the perifollicular ECM in AA with the upregulation of decorin expression by fractional ablative laser alone or combined with other treatments, such as PRP, steroids and vitamin D that is associated with restoration of hair growth in AA. This research carries real hope of recovery for AA sufferers and paves the way for future researches to further explore decorin’s role in AA pathogenesis and to examine the efficacy of decorin-contaning preparations as a treatment for AA.

## Data Availability

The data supporting the results of analyses presented in the paper is available with the corresponding author whenever needed.
